# Bee venom phospholipase A2 ameliorates Alzheimer’s disease pathology in Aβ vaccination treatment without inducing neuro-inflammation in a 3xTg-AD mouse model

**DOI:** 10.1038/s41598-018-35030-1

**Published:** 2018-11-26

**Authors:** Hyunjung Baek, Chan-ju Lee, Da Bin Choi, Nam-sik Kim, Yong-Suk Kim, Young Jun Ye, Youn-Sub Kim, Jin Su Kim, Insop Shim, Hyunsu Bae

**Affiliations:** 1 Department of Physiology, College of Korean Medicine, Kyung Hee University, Seoul, Republic of Korea; 2 Department of Acupuncutre & Moxibustion Medicine, College of Korean Medicine, Kyung Hee University, Seoul, Republic of Korea; 3 Department of Anatomy-Pointology, College of Korean Medicine, Gachon University, Seongnam, 13120 Republic of Korea; 4 Division of RI-Convergence Research, Korea Institute Radiological and Medical Sciences, 75 Nowon-Gil, Gongneung-Dong, Nowon-Gu, Seoul, 01812 Republic of Korea; 5 Acupuncture and Meridian Science Research Center, College of Korean Medical Science Graduate School, Kyung Hee University, Seoul, Republic of Korea

## Abstract

Alzheimer’s disease (AD) is the most common form of dementia and is characterized by an imbalance between the production and clearance of amyloid-beta (Aβ) and tau proteins. Although vaccination against Aβ peptide results in a dramatic reduction in Aβ pathology in experimental mouse models, the initial clinical trial for an active Aβ vaccine was halted early due to the development of acute meningoencephalitis in 6% of the immunized patients, which likely involved a T-cell mediated pro-inflammatory response. In this study, we aimed to determine whether bee venom phospholipase A2 (bvPLA2) treatment would induce Tregs and ameliorate AD pathology without unwanted T cell-mediated inflammation. First, we investigated the effects of bvPLA2 on the inflammatory infiltration caused by Aβ vaccination. Inflammatory aggregates of CD3^+^ T lymphocytes and macrophages were found in the brains and spinal cords of mice treated with Aβ. However, administration of bvPLA2 dramatically eliminated central nervous system inflammation following Aβ immunization. In AD model mice (3xTg-AD mice), bvPLA2 administration significantly ameliorated cognitive deficits and reduced Aβ burdens in the brains of Aβ-vaccinated 3xTg-AD mice. Additionally, we examined brain glucose metabolism using positron emission tomography with ^18^F-2 fluoro-2-deoxy-d-glucose. Cerebral glucose uptake was considerably higher in the brains of Aβ-vaccinated 3xTg-AD mice that received bvPLA2 than those that did not. The present study suggests that the modulation of Treg populations via bvPLA2 treatment may be a new therapeutic approach to attenuate the progression of AD in conjunction with Aβ vaccination therapy without an adverse inflammatory response.

## Introduction

Alzheimer’s disease (AD) is a severe neurodegenerative disorder characterized by the accumulation of two hallmark proteins, amyloid-β (Aβ) peptides and neurofibrillary tangles, that play key roles in neuroinflammation, including the production of pro-inflammatory cytokines and activation of microglial cells and complements. According to the “amyloid cascade hypothesis,” deposition of Aβ peptide in amyloid plaques may cause deleterious events, such as neurofibrillary tangle formation, neuronal dysfunction, and death^1–3^. The present treatments available for AD patients are limited to symptomatic management that consists mostly of acetylcholinesterase inhibitors and an *N*-methyl-d-aspartate receptor antagonist. Thus, the Aβ pathway has become a major target for the development of disease-modifying drugs that stabilize or slow the AD pathological process.

Aβ immunotherapy involves lowering the production of neurotoxic oligomeric precursors consisting of aggregated Aβ or increasing the rate of Aβ clearance from the brain^4,5^. Active immunization following vaccination against Aβ1-42 peptide was reported to have a therapeutic effect in a murine model of AD^6,7^. In view of these encouraging results, a clinical Aβ vaccination trial (AN1792) was initiated, and modest beneficial effects were observed in a cohort of Aβ1-42-immunized AD patients. However, the trial was interrupted due to the development of meningoencephalitis in 6% of the patients, revealing the presence of autoreactive T cell responses^8^. To avoid a T cell-mediated immune response after active vaccination, current alternative vaccination strategies are focused on generating epitope-specific Aβ peptides.

CD4^+^CD25^+^Foxp3^+^ regulatory T cells (Tregs) comprise a functionally distinct subset of mature T cells that act as the master regulators in the development and maintenance of immune tolerance^9,10^. Tregs suppress a variety of immune cells, including B cells, natural killer (NK) cells, CD4^+^ or CD8^+^ T cells, and both monocytes and dendritic cells. Naturally occurring Tregs (nTregs) originate from the thymus and constitute a critical arm of the active mechanisms of peripheral tolerance, particularly to self-antigens. In contrast, inducible Tregs (iTregs) originate from the periphery, mainly from CD4^+^ T cell activation by specific target antigens presented by antigen-presenting cells (APC) in the presence of specific immune regulatory cytokines (essentially TGF-β). Following adequate antigen stimulation in the presence of cognate antigen and specialized immune-regulatory cytokines, iTregs differentiate into CD25^+^ and Foxp3^+^ Tregs. Recently, adoptive therapy of antigen-specific iTregs targeted to allergy, asthma, and autoimmune disease antigens holds promise as an alternative to an immunomodulatory strategy^11–13^. A growing body of evidence suggests that the modulation of Treg function may be a potential therapeutic approach for neuroprotection in various neurodegenerative disorders^14,15^. A better understanding of Tregs may therefore facilitate the development of new alternative strategies for modulating the inflammatory responses involved in AD.

We have previously reported the protective role of Tregs against AD progression^16^. Adoptive transfer of Tregs improved cognitive deficits and reduced cortical and hippocampal Aβ plaque deposition in a mouse model of AD (3xTg-AD). Furthermore, depletion of Tregs resulted in marked aggravation of the spatial learning deficits and a decrease in glucose metabolism. We also reported that bee venom phospholipase A2 (bvPLA2) treatment exerted neuroprotective effects in a mouse model of AD via microglial inactivation and a reduction in CD4^+^ T cell infiltration^17^. Therefore, in the present study, we aimed to determine whether bvPLA2 treatment would induce Tregs and ameliorate AD pathology without unwanted T cell-mediated inflammation following Aβ vaccination. Our findings demonstrate that bvPLA2-mediated expansion of Tregs together with co-immunization of Aβ42 vaccine may have distinct effects on immunosuppressive responses, facilitating a balance in homeostasis in the context of neurodegenerative diseases, including AD. bvPLA2-mediated induction of Tregs improved cognitive function, reduced Aβ deposition and the production of inflammatory cytokines, and increased glucose uptake in Aβ-vaccinated 3xTg-AD mice. We also found that bvPLA2-induced Tregs significantly reduced inflammatory infiltration in the brain and spinal cord following Aβ immunization and injection of pertussis toxin (PT) as a model for neuro-inflammation. These findings suggest that Treg induction by bvPLA2 treatment may improve the safety of Aβ-based vaccines against central nervous system (CNS) inflammation. Thus, bvPLA2 treatment with Aβ vaccine may be a therapeutic strategy to induce Tregs targeted against Aβ42 antigen without adverse neuro-inflammation.

## Results

### bvPLA2 attenuated aberrant autoimmune reactions against Aβ peptide leading to neuro-inflammation in C57BL/6 mice

**Figure 1:**
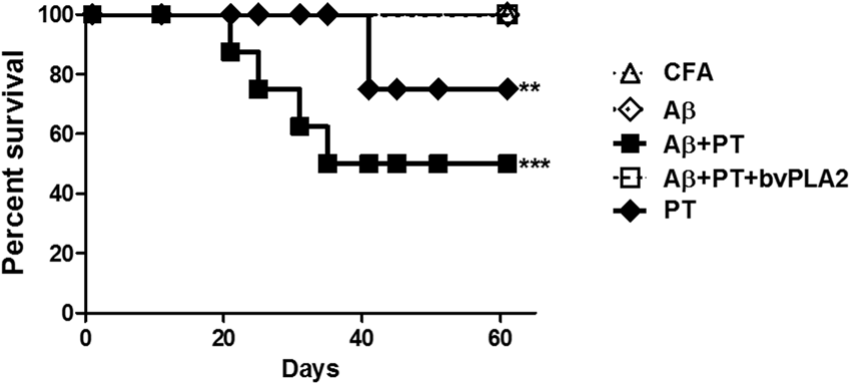
Time course of the survival rates of mice treated with bvPLA2 followed by Aβ immunization with or without PT. To induce a model of neuro-inflammation in C57BL/6 mice, the antigen suspension was mixed with 1:1 with CFA, and 100 µg of the Aβ1-42 peptide was injected subcutaneously. 500 ng of PT was intravenously administered the same day and 48 h later Aβ immunization. 0.5 mg/kg of bvPLA2 was given to mice after Aβ immunization. Mice were monitored for survival over 60 days. The survival rate was evaluated using Kaplan-Meier curves. **P < 0.01 and ***P < 0.01 vs. Aβ + PT + bvPLA2.

**Figure 2:**
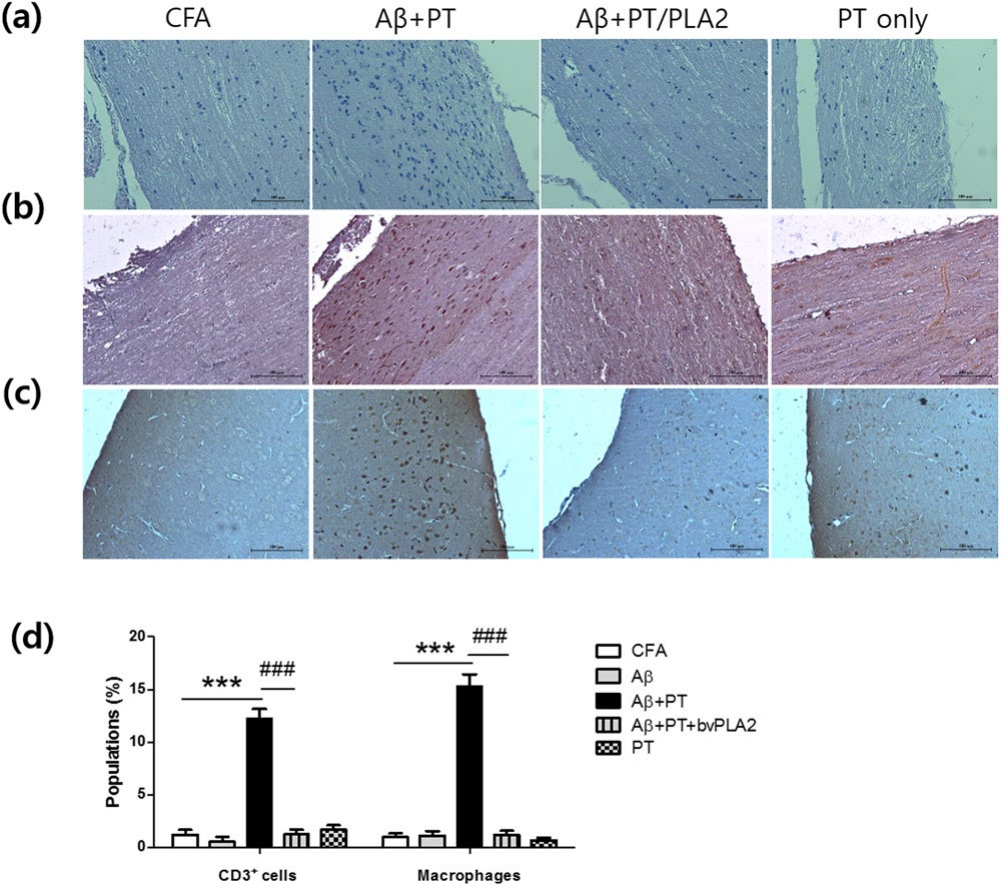
Effects of bvPLA2 on inflammatory infiltration in the brains and spinal cords of C57BL/6 mice following Aβ immunization with or without PT. (**a**) The marginal region of spinal cord tissues was stained with hematoxylin and eosin (H&E) for analysis of cellular infiltration. Tissues were stained with (**b**) anti-CD3 antibody and (**c**) biotin-conjugated BS-I isolectin B4 for T cell and macrophage infiltration, respectively. Figure is representative of sections from five individual mice. The marginal region of spinal cord and the cortex region of brain were depicted. Scale bars 100 µm.

First, we examined the effect of bvPLA2 treatment on adverse neuro-inflammations against full-length Aβ peptide vaccination. To boost the T cell response against Aβ, C57BL/6 mice were injected with 100 µg Aβ1-42 peptide and boosted intravenously twice with 500 ng pertussis toxin (PT) for two consecutive days. PT is widely used to facilitate the induction of experimental autoimmune encephalomyelitis (EAE) in rodents because of its ability to open the blood-brain barrier (BBB). As expected, none of the mice immunized with Aβ alone showed any abnormality up to 60 days after vaccination. Conversely, the survival rate decreased to 60% after immunization with PT treatment. In contrast, all mice treated by bvPLA2, Aβ, and PT alone survived and showed no abnormal symptoms (Fig. 1). Inflammatory infiltration and aggregates of CD3^+^ T lymphocytes and BS-I isolectin B4^+^ macrophages were investigated in the brain and spinal cord tissues. In all mice treated with Aβ and PT, inflammatory infiltration was observed and was mainly localized around the marginal zone of the spinal cord, while this was not seen in the Complete Freund’s Adjuvant (CFA) group (Fig. 2(a)). Infiltration of inflammatory cells was also not observed in the Aβ + PT + bvPLA2 group (Table 1 and Fig. 2(b)). Inflammatory aggregates of macrophages and CD3^+^ T lymphocytes were found in the brains and spinal cords of mice treated with Aβ and PT (Fig. 2(b). In contrast, CD3^+^ T lymphocytes were diminished in bvPLA2-treated mice administered Aβ and PT (Fig. 2(c) and Table 1). The relative number of macrophages was about 25-fold lower in the Aβ + PT + bvPLA2 group than those in the Aβ + PT groups (Table 1). These results suggest that the induction of Tregs by bvPLA2 treatment suppresses effector T cells and macrophages in the pathogenesis of Aβ vaccination-induced neuro-inflammation.

**Table 1:** Disease features of C57BL/6 mouse groups immunized with amyloid-β with or without PT.

Group	Antigen	PT	bvPLA2	Survival rate (%)	Inflammatory infiltration	CD3^+^ cells	Macrophages
1	—	—	—	10/10 (100)	0.1 ± 0.3	1.23 ± 0.8	0.46 ± 1.2
2	Aβ1-42	No	No	10/10 (100)	0.13 ± 0.3	0.25 ± 1.2	0.61 ± 1.4
3	Aβ1-42	Yes	No	6/10 (60)	18.9 ± 1.9	12.21 ± 1.6	15.3 ± 2.0
4	Aβ1-42	Yes	Yes	10/10 (100)	0.37 ± 0.1	1.25 ± 0.8	1.22 ± 0.6
5	—	Yes	No	9/10 (90)	0.14 ± 0.6	1.7 ± 0.7	0.7 ± 0.4

### bvPLA2 treatment significantly alleviated memory impairments in Aβ-vaccinated 3xTg-AD mice

**Figure 3:**
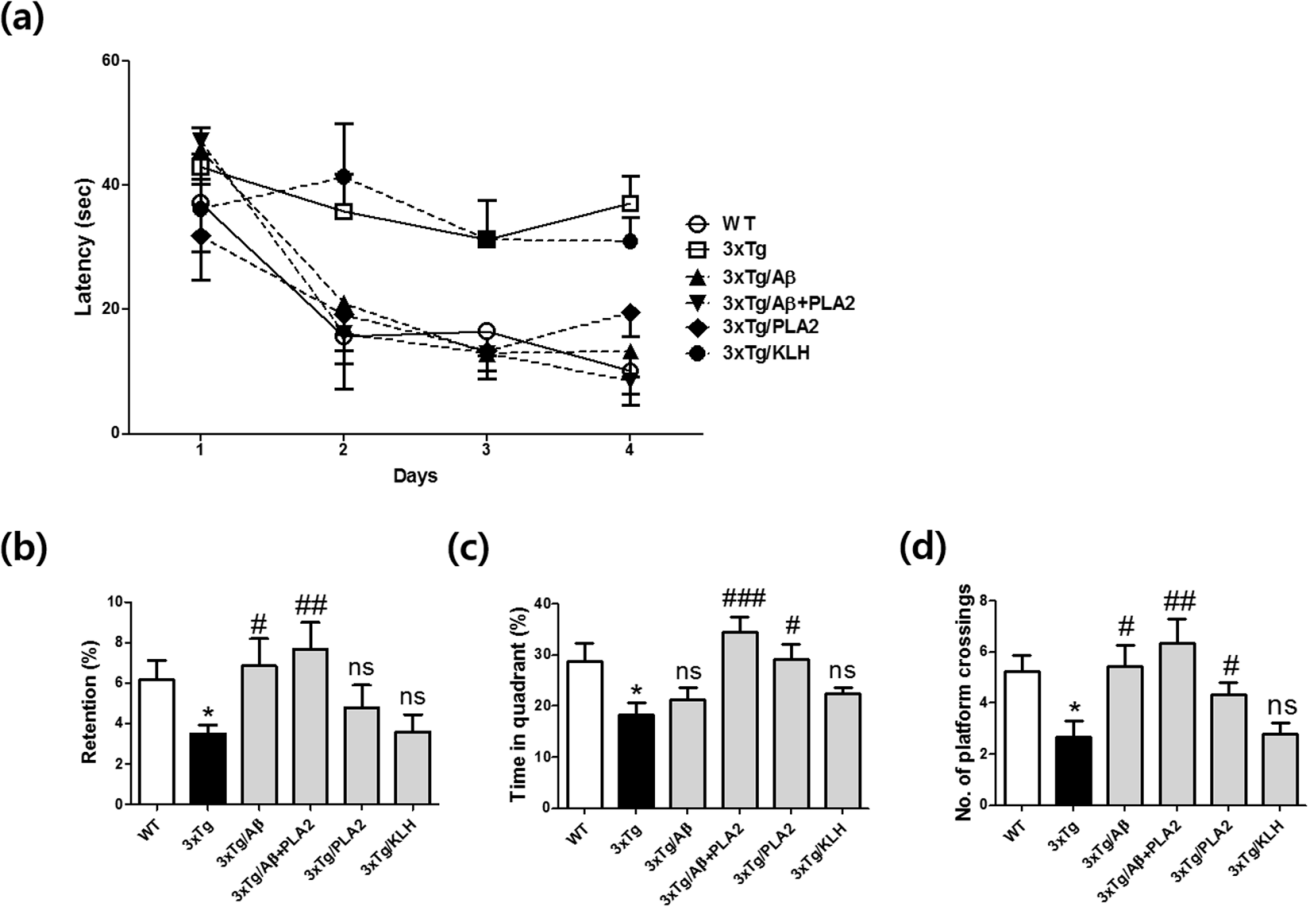
Effects of bvPLA2 on spatial learning and memory in Aβ-vaccinated 3xTg-AD mice. 3xTg-AD mice were tested after 3 months of Aβ1-42 peptide immunization with or without bvPLA2 injection. Mice were subjected to the MWM. Training trials (60 s each) were performed three times a day for 4 days for the acquisition test. In the retention test, the mice were submitted to a probe trial in which the platform was removed from the pool. (**a**) Escape latency, (**b**) retention, (**c**) time in the target quadrant, and (**d**) number of platform crossings. The data are shown as means ± SEM. *P < 0.05 vs. WT; ^#^*P* < 0.05, ^##^*P* < 0.01, and ^###^*P* < 0.001 vs. 3xTg.

To evaluate the neuroprotective effects of bvPLA2 in AD model mice undergoing Aβ vaccination therapy, mice were immunized with Aβ1-42 peptide six times over 3 months, and bvPLA2 was injected intraperitoneally once a week during the Aβ immunization period. We used the morris water maze (MWM) to examine the psychological process and neural mechanisms of spatial learning and memory in 3xTg-AD mice, which is placed in a large circular pool of water and required to escape from water onto a hidden platform. Mice relies on distal visual cues to navigate from starting locations around the testing area to a submerged escape platform. The latency times taken to reach the hidden platform were measured during the acquisition trials (Fig. 3(a)). On the 4th day, the latency times of the 3xTg/Aβ + PLA2 group were similar to that those of the WT group, whereas the 3xTg and 3xTg/KLH (keyhole limpet hemocyanin) groups exhibited the longest latency times. The number of times the mice traveled into the location where the hidden platform was previously placed was significantly greater in the 3xTg/Aβ + PLA2 group than in the 3xTg/Aβ group (Fig. 3(b)). The time spent in the target quadrant and the number of platform crossings were both dramatically increased in the 3xTg/Aβ + PLA2 group compared with those in the 3xTg/ Aβ group (Fig. 3(c,d)). KLH-treated transgenic mice failed to demonstrate learning or memory of the platform location. These data indicate that bvPLA2 treatment significantly reverts the memory deficits seen in Aβ-vaccinated 3xTg-AD mice.

### Effects of bvPLA2 on inflammatory cytokine production in Aβ-vaccinated AD model mice

**Figure 4:**
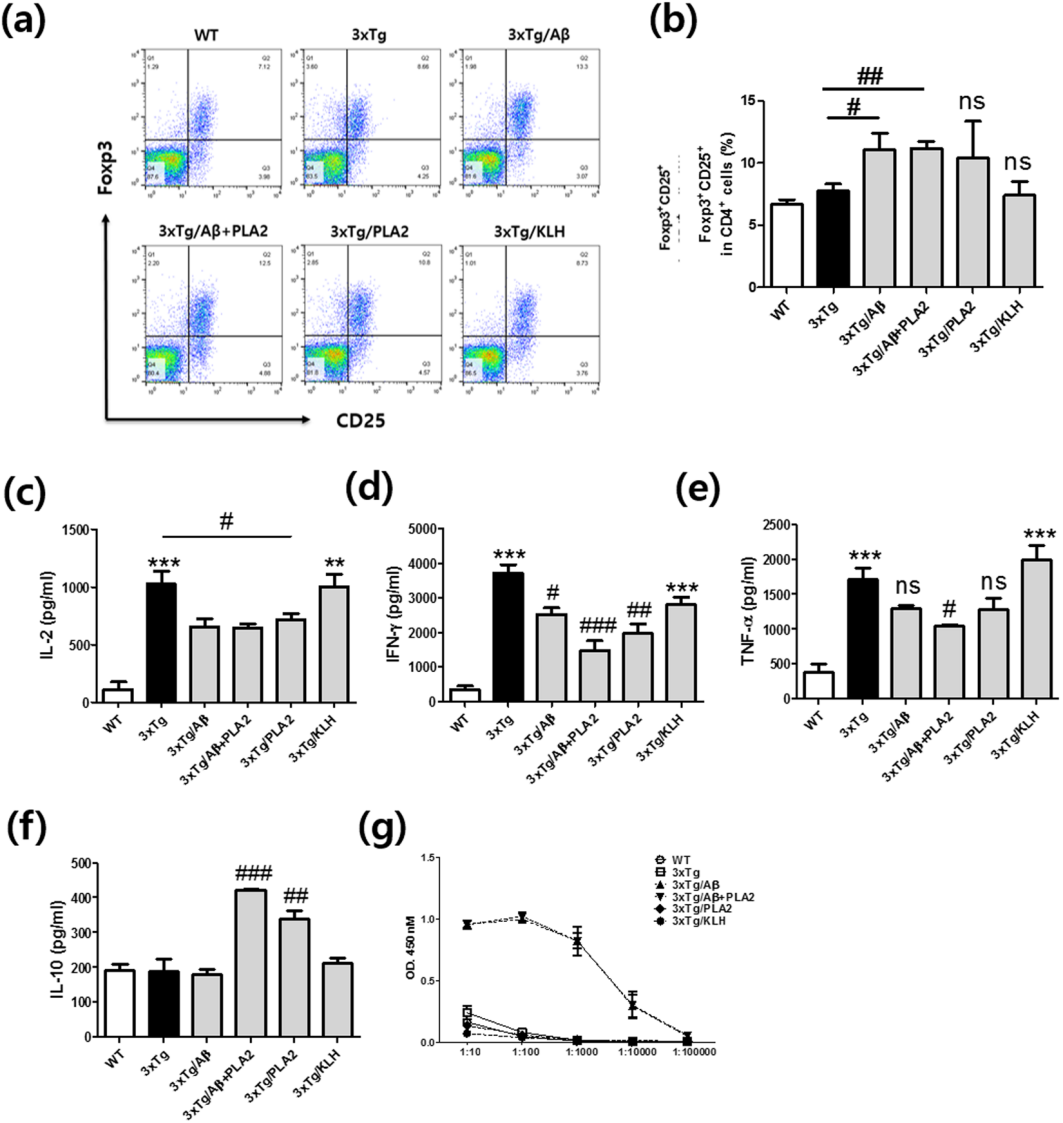
Effects of bvPLA2 treatment on Tregs, inflammatory cytokine production, and serum anti-Aβ antibody levels in Aβ-vaccinated 3xTg-AD mice. (**a**) Dot plots of CD4^+^CD25^+^Foxp3^+^ populations were determined among the splenocytes of each group. (**b**) Percentages of CD4^+^CD25^+^Foxp3^+^ cells. (**c**) IL-2, (**d**) IFN-γ, (**e**) TNF-α, and (**f**) IL-10 were measured using the CBA mouse Th1/2/17 cytokine kit. (**g**) Serum levels of anti-Aβ antibodies were measured using an enzyme-linked immunosorbent assay (ELISA) in which 96-well plates were coated with Aβ1-42 peptide and incubated with each diluted serum sample. After washing, anti-Aβ titers were detected with specific horseradish peroxidase-conjugated anti-mouse IgG antibodies. Absorbance at 450 nm was measured. The data are shown as the mean ± SEM. **P < 0.01 and ***P < 0.001 vs. WT; ^#^*P* < 0.05, ^##^*P* < 0.01, and ^###^*P* < 0.001 vs. 3xTg; ns: not significant.

The proportion of Tregs was measured from the splenocytes of each group and was significantly increased following Aβ vaccination compared to the proportion in the 3xTg group (Fig. 4(a)). However, no significant differences between the 3xTg/Aβ, 3xTg/Aβ + PLA2, and 3xTg/PLA2 groups were found (Fig. 4(b)). To investigate the levels of immune response mediators, the production of pro-inflammatory cytokines, including interleukin (IL)-2, IL-10, interferon (IFN)-γ, and tumor necrosis factor (TNF)-α, in the supernatants of splenocyte cultures was assessed (Fig. 4(c–e)). Levels of IL-2, IFN-γ, and TNF-α were elevated in 3xTg-AD mice compared with those in WT mice (Fig. 4(c–e)). Administration of bvPLA2 significantly reduced the levels of IL-2 and IFN-γ compared with those in the 3xTg-AD group. The reductions of IFN-γ and TNF-α in the 3xTg/Aβ + PLA2 group were greater than those observed in the 3xTg/Aβ group. In contrast, IL-10 levels were significantly elevated in PLA2-treated mice (Fig. 4(f)). IL-10 is a key cytokine produced by Tregs, and elevated IL-10 levels suggest that Tregs are induced by bvPLA2 treatment.

### Effects of bvPLA2 treatment on serum anti-Aβ Antibodies (Abs)

To determine whether Ab production was directed against Aβ, we collected sera from the mice in each group and measured anti-Aβ Ab titers using direct enzyme linked immunosorbent assay (ELISA) methods. Serological analysis indicated that mice injected with Aβ developed Abs against the Aβ peptide. High titers were found in both the 3xTg/Aβ and 3xTg/Aβ + PLA2 groups immunized against Aβ (Fig. 4(g)). In contrast, there was no anti-Aβ activity in the WT, 3xTg, 3xTg/PLA2, or 3xTg/KLH mice at the tested medium serum dilution of 1:1,000, indicating that 3xTg-AD mice did not spontaneously generate Abs reactive to Aβ. Moreover, these results indicate that bvPLA2 treatment had no influence on the production of Aβ-specific Abs.

### Effects of bvPLA2 administration on cerebral Aβ deposition in Aβ-vaccinated 3xTg-AD mice

**Figure 5:**
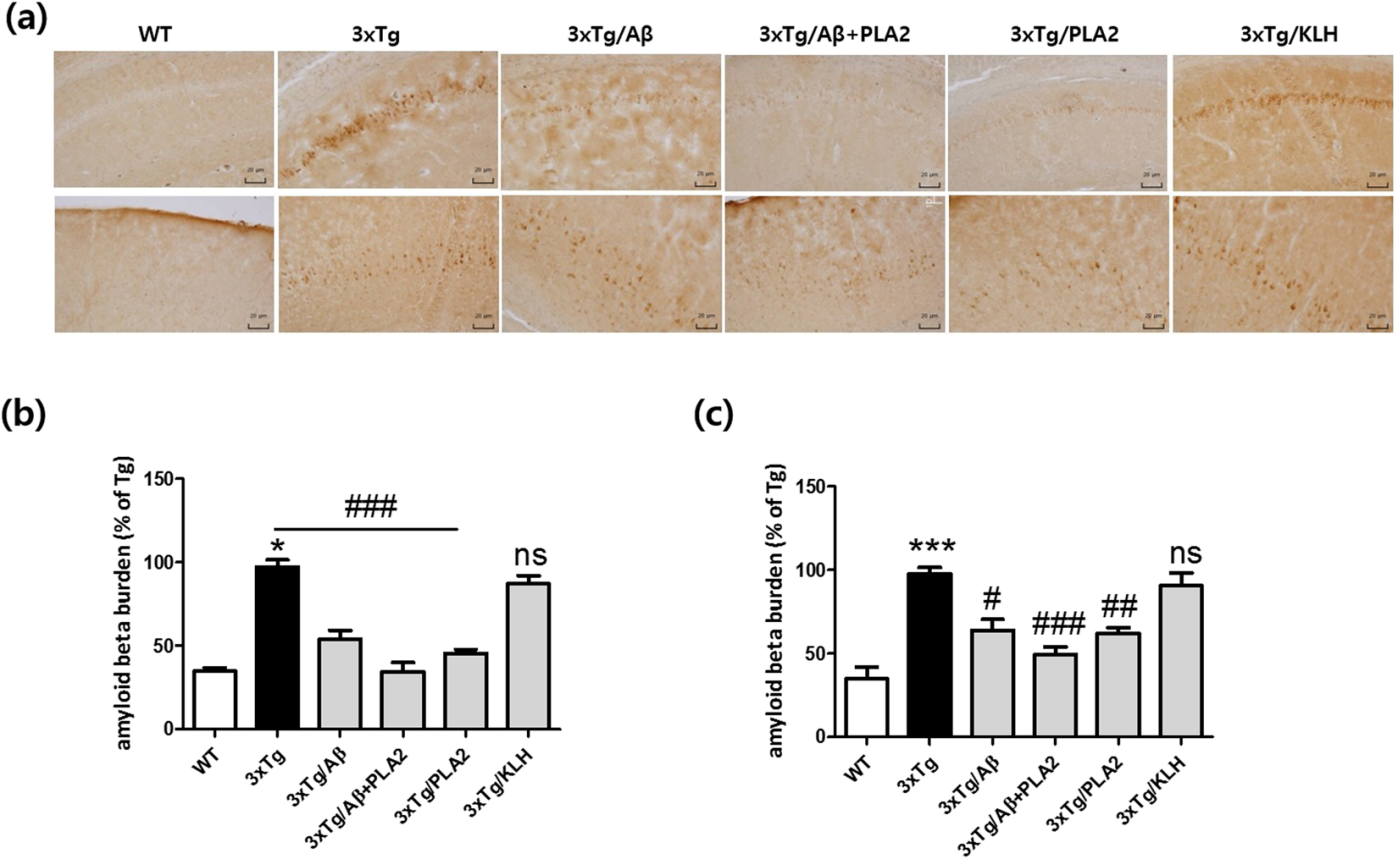
Immunohistochemical staining depicting the effects of bvPLA2 treatment on Aβ burdens in the brains of 3xTg-AD mice. Aβ burdens were determined by immunohistochemical staining with anti-Aβ antibodies. (**a**) Representative images of Aβ staining in the hippocampal CA1 (top) and cortex (bottom) regions. Scale bars 20 µm. Bar graphs of data pertaining to the effects of bvPLA2 treatment on Aβ deposition in (**b**) hippocampal CA1 and (**c**) cortex regions. The data are shown as the mean ± SEM. ***P < 0.001 vs. WT; ^#^*P* < 0.05, ^##^*P* < 0.01, ^###^*P* < 0.001 vs. 3xTg.

The deposition of Aβ significantly increased in the hippocampal CA1 and cortex regions, one of the most plaque-dense areas, in 3xTg-AD mice at the age of 6 months compared with levels in the age-matched WT group (Fig. 5). Immunization with Aβ was associated with a modest reduction in Aβ burden in the frontal cortex, with a significant reduction in the Congo-red stained area of APP/PS1 mice^18^. Similar reductions were observed in the cortex and hippocampus regions in 3xTg/Aβ mice compared with levels in the 3xTg group. Moreover, compared with those in the 3xTg/Aβ group, the Aβ burdens in the hippocampal CA1 and cortex regions were significantly reduced following bvPLA2 treatment. In contrast, there were no significant differences between 3xTg-AD and KLH-treated 3xTg-AD mice.

### Changes in brain glucose metabolism

**Figure 6:**
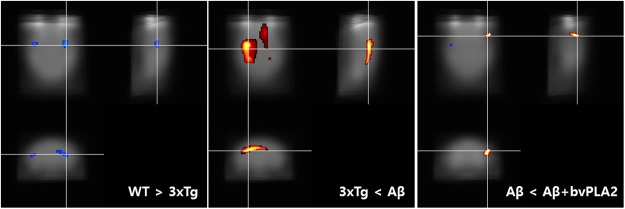
Effect of bvPLA2 on glucose uptake in the brains of 3xTg-AD mice. Voxel-based morphometry revealed right-dominant asymmetry of cerebral glucose metabolism. The result of voxel-based statistical analysis was visualized [^18^F]-FDG scans comparing the WT and 3xTg groups, 3xTg and 3xTg/Aβ groups, and 3xTg/Aβ and 3xTg/Aβ + PLA2 groups. Red indicates a region of relatively high glucose metabolism in comparison to corresponding regions. Blue indicates a region of relatively low glucose metabolism in comparison to corresponding regions.

We determined that bvPLA2 treatment enhanced glucose uptake in the brain using of ^18^F-2 fluoro-2-deoxy-d-glucose ([^18^F]-FDG)-positron emission tomography (PET) imaging. Figure 6 shows coronal and sagittal images of mouse brains depicting [^18^F]-FDG uptake. Scans indicated differences in cerebral glucose metabolism rates between the WT and 3xTg groups, the 3xTg and 3xTg/Aβ groups, and the 3xTg/Aβ and 3xg/Aβ + PLA2 groups (Fig. 6). The uptake of [^18^F]-FDG in 3xTg-AD mice in the region of caudate putamen was significantly decreased compared with that in the WT group (FWE *p* < 0.005). The glucose uptake in the frontal association cortex of the 3xTg/Aβ + PLA2 group was increased compared to that in Aβ-vaccinated 3xTg-AD mice (FWE *p* < 0.005). Decreased glucose uptake in forelimb region (motor associated area) of 3xTg/Aβ + PLA2 group was detected compared to that of Aβ-vaccinated 3xTg-AD mice (FWE *p* < 0.005).

### Effects of Treg depletion on bvPLA2-mediated attenuation of Aβ pathology

**Figure 7:**
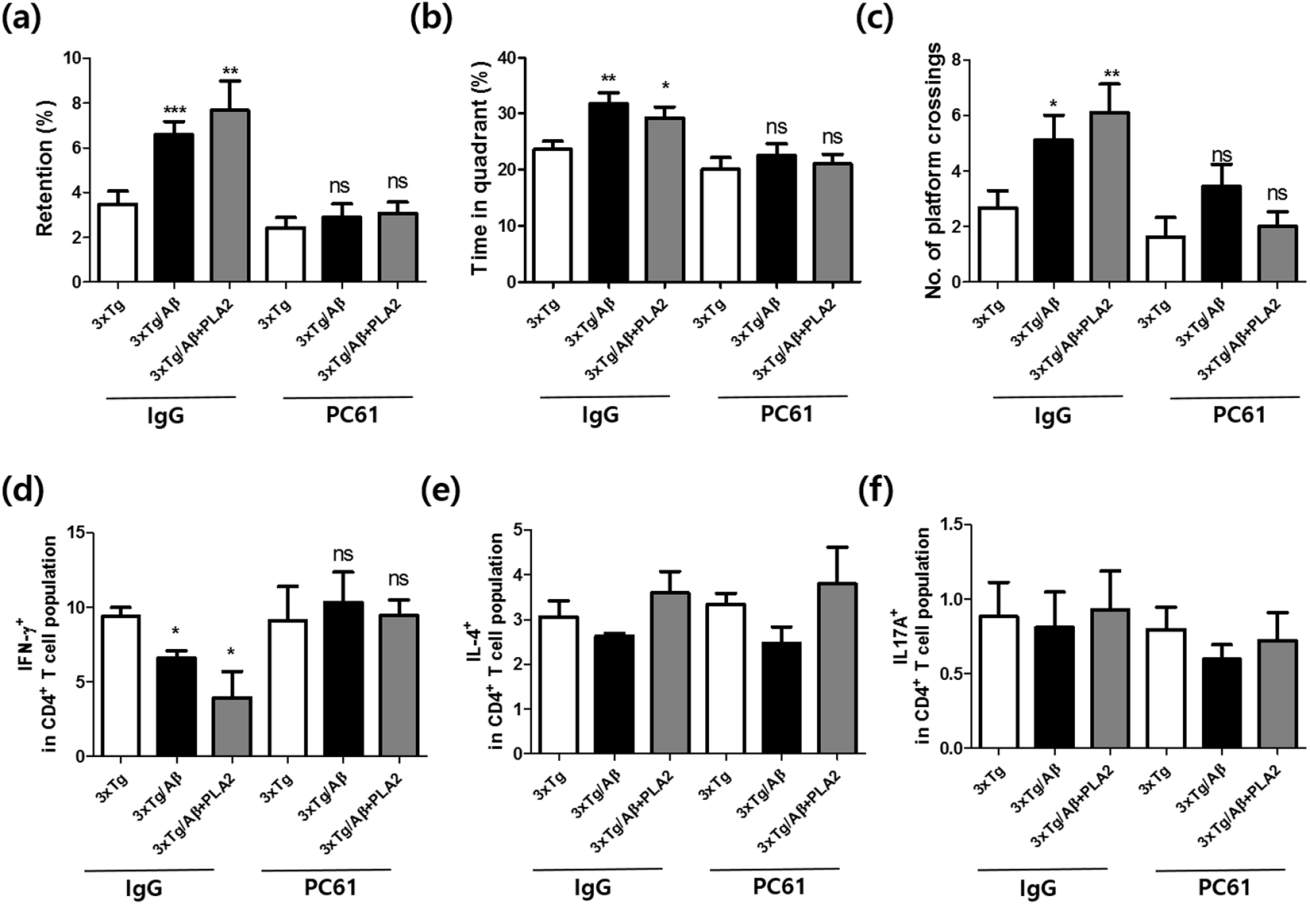
Effects of Treg depletion on bvPLA2-mediated attenuation of Aβ pathology. For Treg depletion, mice received 0.5 mg of rat anti-CD25 IgG (the PC61 group) or total rat IgG (the IgG group) once a week for 3 months. To examine the effects of Treg depletion on bvPLA2-mediated improvements in cognitive deficits, mice were subjected to MWM. (**a**) Retention time in the water maze during the probe trial. (**b**) Time spent in the quadrant in which the platform was placed. (**c**) Crossing times of the platform location. The populations of (**d**) IFN-γ^+^, (**e**) IL-4^+^, and (**f**) IL-17A^+^ cells among CD4^+^ T cells. Data are shown as the means ± SEM. *P < 0.05 and **P < 0.01 vs. 3xTg; ^#^*P* < 0.05, ^##^*P* < 0.01, ^###^*P* < 0.001 vs. the IgG group; ns: not significant.

To provide a more stringent setting in which to test the involvement of Tregs following bvPLA2 treatment, we depleted the Treg population using the PC61 anti-CD25 monoclonal Ab during Aβ vaccination and bvPLA2 treatment. We investigated whether the depletion of Tregs induces altered spatial learning and memory in the MWM. The retention time was dramatically increased in the IgG group, whereas PC61-injected mice showed an inability to find the platform location independent of Aβ and bvPLA2 treatment (Fig. 7(a–c)). Changes in Th1, Th2, and Th17 populations generally suggest an alternative status of the T helper phenotype. Therefore, we quantified IFN-γ-, IL4-, and IL17A-producing CD4^+^ T cells in both IgG- and PC61-injected mice. As shown in Fig. 7(d–f), Th1 populations were decreased in the IgG group compared with those in 3xTg-AD mice. In contrast, Th1 populations in Treg-depleted mice were significantly increased compared with those in the IgG group. Alterations in Th2 and Th17 populations were not observed in any group.

## Discussion

In this study, we hypothesized that the induction of Tregs by bvPLA2 may improve cognitive function and reduce the pathological features of AD in Aβ vaccination treatment. The data reported here support the idea that the use of bvPLA2 in Aβ vaccination treatment may lead to a reduction in aberrant autoimmune reactions against Aβ that provoke CNS inflammation. We found that vaccination with Aβ42 combined with PT triggers an adverse autoimmune response against Aβ, leading to neuro-inflammation. PT has been reported to enhance immune responsiveness, in particular boosting T cell responses and predisposing to autoimmune reactions^19,20^. In this study, administration of bvPLA2 dramatically eliminated inflammatory infiltration, especially of T lymphocytes and macrophages, in the brain and spinal cord tissues compared to that observed in mice that treated with Aβ immunization and with PT. Furthermore, vaccination with Aβ protects transgenic mice from spatial learning deficits and was also associated with a reduction in Aβ deposition in 3xTg-AD mice. Administration of bvPLA2 decreased cognitive deficits and reduced Aβ pathology in the hippocampal and cortex regions of AD mice model compared with those in Aβ-vaccinated 3xTg-AD mice. Treg populations were significantly increased in the Aβ-vaccinated groups. Cerebral glucose uptake was considerably increased in the brains of the bvPLA2-treated group compared to that in the Aβ-vaccinated 3xTg-AD group. However, the enhancement of neuroprotective effects by bvPLA2 treatment was not observed following Treg depletion.

After the failure of the AN1792 trial, several second-generation vaccines were developed using the N-terminal portion of the Aβ peptide to produce Abs without inducing Aβ-specific Th1-mediated cellular immune responses^4,21^. CAD106, containing the Aβ1-6 peptide coupled to a carrier containing 180 copies of bacteriophage QB coat protein, is a new Aβ vaccine in Phase II/III clinical trials^22^. ACC-001 is an Aβ1-7 peptide conjugated with a carrier protein, an inactivated diphtheria toxin. ACC-001 induced a humoral immune response with no sign of side effects in a phase I trial and is currently being evaluated in a phase II trial. Despite decades of extensive efforts to develop Aβ vaccines, there is still no effective therapy available to halt the progressive loss of neurons and deterioration of cognitive faculties in AD patients.

Tregs are essential for the maintenance of self-tolerance and immune homeostasis, which is required for the prevention of autoimmune disease, allergies, and allograft rejection^23^. Tregs normally serve to suppress the systemic immune system and protect against autoimmune disease. However, in AD mouse models, it appears that this immunosuppressive activity may be detrimental, impairing the ability of the adaptive immune system to respond to and restrain Aβ pathology. Therefore, the modulation of Tregs has been proposed as a potential therapeutic target for the treatment of neuroinflammation-mediated diseases, including AD^24^. In a previous report, we showed that the adoptive transfer of CD4^+^CD25^+^ Treg populations dramatically decreased IFN-γ production compared to that in the 3xTg control group^16^. Here, we observed the increased production of IL-10 and a reduction in IFN-γ levels in the bvPLA2-treated groups. This suggests that Tregs, induced by bvPLA2 treatment, secrete the cytokine IL-10, resulting in the suppression of other immune cells, such as the Th1 population.

Several studies have reported that serum levels of anti-Aβ Ab were significantly higher in healthy age-matched controls than in AD patients^25,26^. In contrast, other studies have indicated that anti-Aβ Ab titers are much higher in AD patients than in healthy controls and are negatively correlated with cognitive impairment^27^. In Aβ vaccination therapy, Aβ-specific Abs are efficiently generated in AD patients and murine models treated with the vaccine. However, Hock *et al*. reported no correlation between the incidence of clinical signs and the magnitude of anti-Aβ Ab titers^28^. Two patients with clinical signs of acute meningoencephalitis and who generated Aβ-specific Abs remained cognitively stable 1 year after vaccination, whereas the cognitive function of one patient with acute clinical signs without Abs against Aβ continued to decline following recovery from meningoencephalitis. In this study, we examined the effects of bvPLA2 treatment on the production of Aβ-specific Abs. As expected, bvPLA2 treatment had no effect on the production of Abs against Aβ, whereas serum Aβ Ab titers were elevated in Aβ-immunized mice. These results indicate that the modulation of Tregs by bvPLA2 treatment may affect neuroinflammation without inducing an increase in serum levels of anti-Aβ Ab in response to Aβ vaccination.

In the present study, we represented the effects of bvPLA2 administration on Aβ pathophysiology with combination Aβ peptide vaccination treatment in 3xTg-AD mice. Several studies reported that bvPLA2 can promote Treg cell-mediated anti-inflammatory responses in several animal models of allergic asthma, Parkinson’s disease, and cisplatin-induced acute kidney injury^29–31^. Chung *et al*., found that bvPLA2 directly bound to mannose receptor CD206 on the surface of dendritic cells (DC) and consequently induce the expression of COX-2^29^. Elevated COX-2 promotes the secretion of prostaglandin E2 (PGE2), resulting in Treg differentiation via EP2 receptor signaling^29^. Here, we demonstrated that bvPLA2 treatment can ameliorate Aβ pathology without adverse neuro-inflammation in Aβ-vaccinated 3xTg-AD mice. The neuroprotective and anti-inflammatory effects of bvPLA2 are mediated by IL-10 production and Treg cell modulation. However, the exact mechanisms of bvPLA2 treatment are further to be elucidated.

Taken together, our data highlight the therapeutic potential of Treg modulation via bvPLA2 treatment for Aβ immunotherapy in AD. While therapies targeting a single pathway have limited benefits and may not demonstrate optimal efficacy, combination therapies targeting the Aβ pathway and bvPLA2-mediated immune suppression may ultimately be required.

## Materials and Methods

### Animals

AD model (3xTg-AD) mice harboring transgenes encoding APP (KM670/671NL), PS1 (M146V), and tau (P301L) proteins [B6;129-*Psen1*^*tm1Mpm*^ Tg(APPSwe,tauP301L)1Lfa/J] were obtained from Jackson Laboratory (Bar Harbor, ME, USA). Age-matched male C57BL/6 mice were purchased from Charles River Korea (OrientBio, Sungnam, Korea). All animals were maintained under specific pathogen-free conditions and a 12-hour light/dark cycle. All mice had free access to food and water during the experiments. All animal experiments were conducted in accordance with the Rules for Animal Care and the Guiding Principles for Experiments Using Animals and were approved by the University of Kyung Hee Animal Care and Use Committee [KHUASP(SE)-16-085].

### Aβ vaccination protocols

For Aβ vaccination, 3 month-old 3xTg-AD mice were used. Aβ1-42 peptide (Genescript, Piscataway, NJ, USA) was suspended in 450 µl distilled water (DW), mixed with 50 µl 10× phosphate-buffered saline (PBS) to yield 1× PBS, and incubated overnight (O/N) at 37 °C. The antigen suspension was mixed 1:1 with complete Freund’s adjuvant (CFA), and 100 µg of the Aβ preparation was injected subcutaneously on days 0, 14, 28, 42, 56, and 70. Control mice were injected with PBS or keyhole limpet hemocyanin (KLH) in CFA that was prepared in the same manner. bvPLA2 (Sigma-Aldrich, St. Louis, MO, USA) dissolved in PBS and administered by intraperitoneal injection at a dose of 0.5 mg/kg once a week for 3 months. bvPLA2 injected 3 days after Aβ immunization. Mice were randomly assigned to six groups as follows: (1) PBS-treated wild-type mice (WT); (2) PBS-treated 3xTg-AD mice (3xTg); (3) Aβ-vaccinated 3xTg-AD mice (3xTg/Aβ); (4) bvPLA2-treated and Aβ-vaccinated 3xTg-AD mice (3xTg/Aβ + PLA2); (5) bvPLA2-treated 3xTg-AD mice (3xTg/PLA2); and (6) KLH-treated 3xTg-AD mice (3xTg/KLH). For Treg depletion, mice received a dose of 0.5 mg of rat anti-CD25 IgG (clone PC61) or total rat IgG once a week for 3 months. The rat anti-CD25 IgG was generated in-house from hybridomas obtained from the American Type Culture Collection (Manassas, VA, USA). The efficacy of Treg depletion was analyzed by flow cytometry using phycoerythrin (PE)-labeled anti-CD25 and fluorescein isothiocyanate (FITC)-conjugated anti-CD4 antibodies (Abs). There were five to seven mice per group.

To induce a model of neuro-inflammation in C57BL/6 mice, the antigen suspension was mixed 1:1 with CFA, and 100 µg of the Aβ1-42 peptide was injected subcutaneously. This was followed by intravenous administration of 500 ng of pertussis toxin (PT; Sigma-Aldrich) the same day and 48 h later. Control mice were immunized with CFA alone. Mice were divided into five groups, and bvPLA2 was injected intraperitoneally into mice as follows: (1) CFA-treated control (CFA); (2) Aβ-immunized (Aβ); (3) Aβ-immunized with PT (Aβ + PT); (4) Aβ-immunized with PT and bvPLA2 (Aβ + bvPLA2); and (5) PT-injected (PT). The mouse survival rate was evaluated using Kaplan-Meier curves. Mice used in individual experiments were age-matched, and there were five mice per group.

### Morris water maze

Spatial learning and memory were examined in mice using the Morris water maze (MWM) with minor modifications^32^. Briefly, mice were trained in a circular water maze with a 90-cm diameter (opaque water, 22 ± 2 °C) and a 6-cm hidden platform submerged 1-cm below the water surface. The maximal trial duration was 60 s, with 30 s on the platform at the end of each trial. Each animal was trained for one of the different starting positions and the swimming path once per day for 4 days, with a new platform location used each day. All mice were subjected to three trials per day at intervals of 15 min for 4 consecutive days. For the probe trial, the platform was removed from the pool, and mice were allowed to swim freely for 60 s to search for the previous location of the platform. Escape latency, time spent in the platform quadrant, and number of platform crossings were recorded for each mouse. Data were collected using a video camera connected to a video recorder and a tracking device (S-MART, Pan-Lab, Barcelona, Spain).

### Measurement of Treg populations

Single-cell suspensions of splenocytes were incubated with PE-labeled anti-CD25 and FITC-conjugated anti-CD4 Abs on ice for 30 min. After surface staining, cells were fixed and permeabilized with Perm/Fix solution (eBioscience, San Diego, CA, USA) and stained with PerCP-Cy5.5-conjugated anti-Foxp3 Ab (eBioscience). Stained cells were acquired via BD FACSCalibur flow cytometer (BD Biosciences, San Diego, CA, USA), and the data were analyzed using FLOWJO software (BD Biosciences).

### Measurement of cytokines in splenocyte cultures

Single-cell suspensions of splenocytes were plated in 96-well tissue culture plates in RPMI-1640 medium (WelGENE Inc., Taegu, Korea) supplemented with 10% fetal bovine serum, 50 IU/ml penicillin, and 50 µg/ml streptomycin (Hyclone, Logan, UT, USA). Cultures were activated in the presence of plate-bound anti-CD3 and soluble anti-CD28 Abs (BD Biosciences). Cytokines were detected in the supernatants using a mouse cytometric bead array (CBA) kit (BD Biosciences) in accordance with the manufacturer’s instructions. Briefly, samples were added to a mixture of capture Ab, bead reagent, and PE-conjugated detection Ab. The mixture was incubated at room temperature in the dark and then washed. Data were acquired using a BD FACSCalibur flow cytometer and analyzed using BD CellQuest and BD CBA software (BD Biosciences).

### Th1/2/17 phenotyping

Splenocytes were washed, resuspended in complete RPMI-1640 medium, and transferred to 6-well plates. Cultures were stimulated with 50 ng/ml phorbol myristate acetate (PMA; Sigma-Aldrich) and 1 μg/ml ionomycin (Sigma-Aldrich) for 5 h in the presence of Golgi-stop (BD Biosciences). After 5 h of culture, the contents of the wells were transferred and centrifuged at 300 g for 5 min. Cells were aliquoted into tubes and washed once in stain buffer (BD Bioscience). Cells were incubated with FITC-labeled anti-CD4 Ab for surface staining. After surface staining, cells were fixed and permeabilized with Perm/Fix solution (eBioscience) and stained with PE-conjugated anti-IFN-γ, IL-4, and IL-17A Abs (eBioscience). Stained cells were acquired by BD FACSCalibur flow cytometer, and the data were analyzed using FLOWJO software (BD Biosciences).

### Measurement of Aβ-specific Ab titers

Serum anti-Aβ Ab titers were measured via enzyme-linked immunosorbent assay (ELISA). First, 96-well EIA/RIA plates were coated with 10 µg/ml Aβ1-42 peptide in coating buffer and incubated O/N at 4 °C. Plates were washed with washing buffer (0.05% Tween-20 in PBS) and then blocked for 1 h at 37 °C with 1.5% bovine serum albumin (BSA) in wash buffer. Following blocking, the plate was washed three times, and diluted samples were applied in triplicate and incubated at 37 °C for 1 h. Aβ-specific Ab titers were detected with specific horseradish peroxidase (HRP)-conjugated anti-mouse IgG Abs (R&D Systems, Minneapolis, MN, USA).

### Histological analysis

After the behavioral test, mice were transcardially perfused with saline solution containing 0.5% sodium nitrate and heparin (10 U/ml) and fixed with 4% paraformaldehyde (PFA). Each brain was dissected from the skull, post-fixed O/N at 4 °C, dehydrated in a 30% sucrose solution, and frozen-sectioned on a sliding microtome into 30-μm-thick coronal sections. Slides containing the brain sections were boiled in 0.01 M citrate buffer (pH 6.0) for antigen retrieval, incubated with 3% hydrogen peroxide solution for 10 min to eliminate endogenous peroxidase activity, and then washed with PBS. Aβ burdens were detected using mouse monoclonal 4G8 Ab (BioLegend, San Diego, CA, USA) O/N at 4 °C. Brain sections were washed with PBS, incubated with a biotinylated secondary Ab, and processed with an avidin-biotin complex kit (Vectastain ABC kit; Vector Laboratories, Burlingame, CA, USA). Aβ burdens were visualized via incubation with 0.05% diaminobenzidine-HCl (Vector Laboratories). The labeled tissue sections were then mounted and analyzed under a bright-field microscope (Nikon, Tokyo, Japan). The coronal sections of the hippocampus, starting rostrally from anteroposterior −2.1 mm and continuing to anteroposterior −4.5 mm relative to bregma, were examined at x10 magnification using the IMAGE PRO PLUS system (version 4.0; Media Cybernetics, Silver Spring, MD, USA) on a computer attached to a light microscope (Zeiss Axioskop, Oberkochen, Germany) that was interfaced with a charge-coupled device video camera (Kodak Mega Plus model 1.4 I). To determine the density of the Aβ-immunoreactive staining in the hippocampus, a square frame 500 × 500 μm was placed in the dorsal part of the hippocampus. A second square frame of 200 × 200 μm was placed in the corpus callosum to measure the background.

For neuro-inflammation models in C57BL/6 mice, brains and spinal cords were removed from mice and embedded in paraffin. Then, 5-μm-thick sections were cut from all organs and stained with hematoxylin and eosin (H&E) or processed for detection of CD3-positive cells and macrophages. Briefly, after antigen retrieval and removal of endogenous peroxidase activity with 3% H_2_O_2_, slides were incubated with monoclonal anti-CD3 Ab (BioLegend) and biotin-conjugated BS-I isolectin B4 for macrophage signal O/N at 4 °C. Sections were washed with PBS and incubated with an avidin-biotin complex. The CD3-positive cells and macrophages were visualized via incubation with 3,3-diaminobenzidine (DAB).

### ^18^F-fluoro-2-deoxy-d-glucose micro positron emission tomography scanning

All mice were fasted for 8 h before scanning experiments to increase the uptake of ^18^F-2 fluoro-2-deoxy-d-glucose ([^18^F]-FDG) by their brains^33^. There were five mice per group. Before [^18^F]-FDG uptake, all mice were warmed using a heating pad in a cage in accordance with the previously reported optimized [^18^F]-FDG uptake protocol^34^. A total of 1 mCi of [^18^F]-FDG was injected into the tail vein, and the mice were anesthetized with 2% isoflurane in 100% oxygen (Forane solution; ChoongWae Pharma, Seoul, Korea). A Siemens Inveon positron emission tomography (PET) scanner (Siemens Medical Solutions, Malvern, PA, USA) was used for PET imaging. Transmission PET data were acquired for 10 min using a Co-57 point source with an energy window of 120–125 keV. The transverse resolution used was <1.8 mm at the center^35,36^. After allowing 30 min for tracer uptake, 30 min of PET data was acquired within an energy window of 350–650 keV. The emission list-mode PET data were sorted into 3D sinograms and reconstructed using 3DRP methods. The pixel size of the reconstructed images was 0.15 × 0.15 × 0.79 mm^3^. Attenuation and scatter corrections were performed for all datasets.

### Voxel-based statistical analysis

Voxel-based statistical analysis was performed to compare the cerebral glucose metabolisms of the different groups. The procedure used for the statistical parametric mapping (SPM) analysis of the PET data has been described previously^37,38^. Briefly, for efficient spatial normalization, only the brain region was extracted. A study-specific template was then constructed using all of the datasets. The PET data were spatially normalized onto a mouse brain template and smoothed using a Gaussian kernel. Count normalization was performed. A voxel-wise *t*-test comparing the datasets from the various groups was performed using the Statistical Parametric Mapping 5 program (FWE *P* < 0.005, *K* > 50).

### Statistical analysis

All values are presented as the mean ± standard error of the mean (SEM). The statistical significance of each variable was evaluated by one-way analysis of variance (ANOVA) followed by Tukey’s multiple comparison test using Prism 5.01 software (GraphPad Software Inc., San Diego, CA, USA). All experiments were performed in a blinded manner and repeated independently under identical conditions. Differences with *P*-values < 0.05 were considered statistically significant.

## Data Availability

No datasets were generated or analyzed during the current study.
